# Pregnancy intentions and outcomes among young married women in Nepal

**DOI:** 10.1016/j.xagr.2024.100403

**Published:** 2024-10-09

**Authors:** Aimee J. Lansdale, Mahesh C. Puri, Nadia Diamond-Smith

**Affiliations:** 1 Department of Epidemiology and Biostatistics, University of California, San Francisco, CA (Lansdale and Diamond-Smith); 2 Center for Research on Environment Health and Population Activities (CREHPA), Kathmandu, Nepal (Puri)

**Keywords:** pregnancy, pregnancy intention, pregnancy outcome, unintended, Nepal, reproductive health, barriers, contraception

## Abstract

**Background:**

Approximately 44% of Nepalese women ages 15–49, desiring to avoid pregnancy, do not use modern contraceptives, resulting in an estimated 539,000 unintended pregnancies annually.

**Objectives:**

This study aims to investigate the association between young, newly married women's pregnancy intentions and subsequent pregnancies.

**Study design:**

Data were collected longitudinally from 200 recently married women ages 18–25 in Nepal. Surveys conducted every six months over 18 months covered various health domains. The study used mixed-effects logistic regression models to account for repeated measurement of correlated data over time. The primary outcome was pregnancy. Pregnancy intention was determined based on responses to, “When would you like to have a child in case you were to have one?” Participants were recategorized into a dichotomous variable for analytical purposes: “Right away” or “Not right away.”

**Results:**

A total of 133 participants became pregnant during the study, with sociodemographic characteristics showing minimal differences between pregnant and nonpregnant groups. Women intending to become pregnant right away had significantly higher odds of becoming pregnant (OR, 4.03; 95% CI, 2.51–6.48) after adjusting for covariates. Among those not intending immediate pregnancy, over 70% became pregnant, suggesting potential barriers to achieving reproductive goals.

**Conclusions:**

Young, newly married women in Nepal intending immediate pregnancy have higher odds of becoming pregnant. However, a substantial proportion of those hoping to delay pregnancy still experience unintended pregnancies, indicating challenges in meeting reproductive goals. The findings underscore the need for addressing barriers to contraceptive access and societal norms impacting women's reproductive autonomy in Nepal.

## Highlights

• To better understand how women's pregnancy intentions relate to their pregnancies at a future standpoint in Nepal.
• After adjusting for covariates, women intending to become pregnant right away had significantly higher odds of becoming pregnant. Over 70% of women who were not intending immediate pregnancy became pregnant.
• Using longitudinal data, this study adds missing evidence on measuring changing intentions to the current literature which is limited by studies using cross-sectional data or longitudinal data with only two time points.

## Introduction

Approximately 44% of women in Nepal of reproductive age (15–49 years) who want to avoid a pregnancy are not using modern contraceptives.^1^ According to 2017 data, Nepalese women experience an estimated 539,000 unintended pregnancies each year, likely related to this unmet need for contraception.^1^ Unwanted pregnancies and births may have health, economic, and social consequences for women and their families.^2^^,^^3^ For example, unintended pregnancies are associated with unsafe abortions and postpartum depression.^2^^,^^4^^,^^5^

In Nepal there is significant familial emphasis on a woman's fertility such that her ability to conceive and bear children is frequently associated with her intrinsic worth.^6^ This is intertwined with considerable gender inequality, which is perpetuated within households and in the community. Young women who have recently married face a disproportionately low status within the household, placing them at a heightened vulnerability to adverse health consequences.^7^ In addition, the presence of mothers-in-law in this study is significant as in-laws often play substantial roles in household and fertility decision-making.^8^ Combining these factors, young, newly married women have historically faced pressure to bear children soon after marriage to “prove” their fertility.^9^, ^10^, ^11^ However, qualitative evidence from Nepal suggests that newly married women (and their husbands) often want to delay the first birth.^12^ Cultural barriers due to patriarchal norms have shown to limit women's access to contraceptive or legal abortion services, and thus, young couples may struggle to exert reproductive autonomy.^13^^,^^14^ Limited attention has been given to researching the autonomy in reproductive health of this marginalized population.

Information on unintended pregnancies can indicate gaps in reproductive healthcare. However, the measure of unintended pregnancy is complicated by lack of longitudinal data and related biases, including ex post hoc rationalization, where individuals may retrospectively alter their perceptions or justifications of their pregnancy intentions. In this study, we investigated the association between young, newly married women's pregnancy intentions and their subsequent pregnancies. This focus is critical given the limited research on individuals’ pregnancy intentions and outcomes among women in low-income countries (LICs), with most existing evidence coming from cross-sectional studies.^15^ There is also limited data on young newly married women, with most studies focusing on women 15/18–49 years old.

## Materials and methods

We investigated the association between individuals’ pregnancy intentions and their subsequent pregnancies using a longitudinal dataset of newly married women in Nepal who were not pregnant at baseline. In 2018, this study began gathering data from 200 recently married women ages 18–25 years living in Nawalparasi, Nepal. Greater details about the study can be found elsewhere, but we summarize the study.^16^ To be eligible for participation, women had to be married within the last four months, ages 18–25 years, and living with their mothers-in-law. Staff members trained by the Center for Research on Environment Health and Population Activities (CREHPA) approached eligible women to inform them about the study. The participants provided written consent for participating. The study team provided participants with an equivalent of US$3 per visit, in line with local incentive standards. Incentives were based on the expertise of the local study partners. We obtained ethical approval from the institutional review boards at the Nepal Health Research Council (Ref 385/2016, December 8, 2016) and the University of California, San Francisco (Ref 176007, October 10, 2016).

The participants were surveyed at the beginning of the study, which occurred within four months of marriage. Subsequently, they were surveyed every six months for an additional three rounds (total of 18 months). Demographic characteristics for the participants were evaluated across pregnancy using chi-squared tests of trends.

The primary outcome measure was pregnancy. Participants were asked if they were currently pregnant at each interview and responded “Yes” or “No”. We use pregnancy rather than birth because this captures more respondents (the difference between these two indicators is only due to follow up time, where not all women were followed long enough for their baby to be born). The primary predictor of interest was pregnancy intention. Participants were asked, “When would you like to have a child in case you were to have one?” The participant responded with the following options: (1) “When God wants;” (2) “Right away;” and (3) “X years from now,” where X could be any number. This variable was recategorized into a dichotomous variable: (1) Right away and (2) Not right away. Right away included women who had chosen “When God wants” and “Right away” responses, while not right away included women who had indicated they would like to wait at least one 1 year.

To examine the association of pregnancy intention on pregnancy, we estimated the odds ratio (OR) using a mixed effects logistic regression model to account for repeated measurement of correlated data over time. We do this because pregnancy intentions can change over time, and thus, studies with repeated measures of intention alongside pregnancy can help us understand how intentions change and the true burden of unintended pregnancy.^17^^,^^18^ We then used a multivariable mixed effects logistic regression model to adjust for potential confounders. We excluded individuals who had been pregnant before the study began and who were pregnant at baseline. In a sensitivity analysis, we used univariate and multivariate logistic regression models expressed as ORs to see if the results differed when looking at intention at baseline and the pregnancy 2 years later without considering repeated measurements. In this sensitivity analysis, we kept the pregnancy intention response from baseline constant to see if women who said they wanted a baby right away at the beginning of the study were more or less likely to be pregnant during the study period in comparison to those who said they wanted to delay becoming pregnant.

The potential confounders and determination of cutoff points for categorizing continuous variables were derived from published literature.^6^ The time-invariant characteristics, measured at baseline, included participant's age at marriage (continuous), education level (continuous), birth country (Nepal or India), caste (classified as Brahmin or Chhetri, indigenous groups, or so-called untouchables or religious minority group), religion (Hindu or other), wealth (categorized into quintiles), whether a woman's family paid a dowry during marriage (yes or no), partner's age at marriage (continuous), partner's education level (continuous), and marriage type (distinguishing between love and arranged marriage). The time-varying covariates encompassed engaging in paid work in the previous year (yes or no), currently living with their spouse (yes or no), and an empowerment level variable which was created by aggregating participants' responses to questions regarding gender norms, violence, and sexual relationships. Women's empowerment was measured using a set of variables about women's mobility, household decision-making, freedom from family domination, and economic security. A scale was created summing women's empowerment across these domains, and then, due to low empowerment, a binary variable was created with those who had any form of empowerment (score 1 or more) coded as “empowered” and the rest as not empowered.

STATA (version 17.0; StataCorp, College Station, TX) was used for these analyses. An alpha level of <0.05 was considered statistically significant.

## Results

Out of 200 women, 133 became pregnant at one point in time during the study of which 103 gave birth by the end of the study. The sociodemographic characteristics by pregnancy are displayed in Table 1. Most participants completed 6–12 years of formal education, with more than 70% of each group in this education level. Most women were born in Nepal and were Hindu. The mean age, various caste groups, wealth quintile, whether the women's family paid dowry, spouse's education, living with spouse, marriage type, work, and empowerment characteristics were relatively similar between the groups.

**Table 1:** Sociodemographic characteristics by pregnancy and by pregnancy intention (“right away” vs. “not right away”)

	Not pregnant by endline (*N*=38)	Pregnant by endline (*N*=133)	Pregnancy Intention: right away (*N*=67)	Pregnancy Intention: not right away (*N*=116)
Age (years)	21 (1.8)	20 (2.0)	20 (2.0)	21 (2.0)
Years of formal education				
<6 years	4 (10.5)	21 (15.8)	15 (22.4)	10 (8.6)
6–12 years	28 (73.7)	95 (71.4)	47 (70.1)	81 (69.8)
Over 12 years	6 (15.8)	17 (12.8)	5 (7.5)	25 (21.6)
Country of birth				
Nepal	29 (85.3)	86 (71.7)	45 (68.2)	79 (79.0)
India	5 (14.7)	34 (28.3)	21 (31.8)	21 (21.0)
Caste				
Brahmin/Chheetri	13 (34.2)	25 (18.8)	9 (13.4)	35 (30.2)
Indigenous	17 (44.7)	75 (56.4)	35 (52.2)	61 (52.6)
So-called untouchables and religious minority group	8 (21.1)	33 (24.8)	23 (34.4)	20 (17.2)
Religion				
Hindu	32 (84.2)	116 (87.2)	57 (85.1)	102 (87.9)
Other	6 (15.8)	17 (12.8)	10 (14.9)	14 (12.1)
Wealth quintile				
1	6 (15.8)	25 (18.8)	17 (25.4)	15 (12.9)
2	5 (13.2)	31 (23.3)	18 (26.9)	18 (15.5)
3	8 (21.1)	30 (22.6)	14 (20.9)	24 (20.7)
4	11 (28.9)	28 (21.1)	14 (20.9)	29 (25.0)
5	8 (21.1)	19 (14.3)	4 (6.0)	30 (25.9)
Women's family paid dowry during marriage				
No	6 (15.8)	19 (14.3)	6 (9.0)	24 (20.7)
Yes	32 (84.2)	114 (85.7)	61 (91.0)	92 (79.3)
Spouse education				
<6 years	5 (14.3)	11 (8.7)	11 (16.9)	5 (4.7)
6–12 years	24 (68.6)	97 (76.4)	51 (78.5)	74 (69.8)
12+ years	6 (17.1)	19 (15.0)	3 (4.6)	27 (25.5)
Living with spouse				
No	5 (13.2)	8 (6.0)	4 (6.0)	10 (8.6)
Yes	33 (86.8)	125 (94.0)	63 (94.0)	106 (91.4)
Marriage type				
Love	12 (31.6)	36 (27.1)	16 (23.9)	35 (30.2)
Arranged	26 (68.4)	97 (72.9)	51 (76.1)	81 (69.8)
Work (paid)				
No	31 (81.6)	96 (72.2)	54 (80.6)	80 (69.0)
Yes	7 (18.4)	37 (27.8)	13 (19.4)	36 (31.0)
Empowerment level (baseline)				
Not Empowered	12 (31.6)	62 (46.6)	38 (56.7)	39 (33.6)
Empowered	26 (68.4)	72 (53.4)	29 (43.3)	77 (66.4)

Data are presented as mean (SD) or number (percentage).

Table 1 presents the social and structural characteristics of women by pregnancy intention. Women who did not intend on becoming pregnant immediately had a higher percentage who had completed over 12 years of education, were in the 5th wealth quintile, had families who did not pay a dowry, had a spouse who had completed over 12 years of education, worked for pay, and felt empowered. The proportion of religion types, those living with their spouse, and different marriage types (love vs. arranged) were similar among the groups.

**Figure 1:**
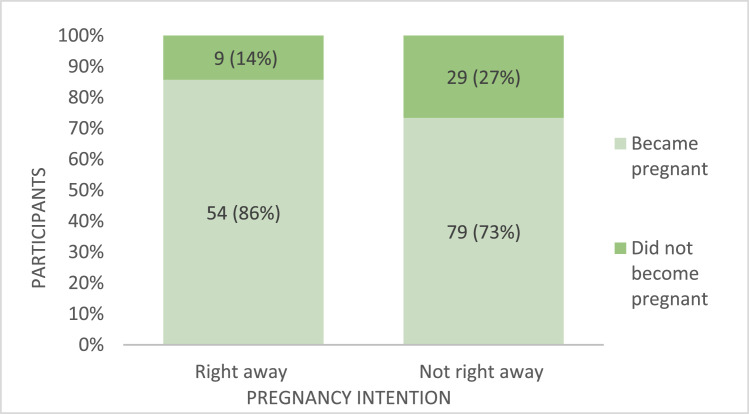
Proportion of pregnancies by pregnancy intention

The proportion of pregnancies by intention after excluding women pregnant at baseline (*n*=15) and women who had been pregnant before the study began (*n*=1) is presented in Figure 1. Among those who said their intention was to become pregnant right away, 85.71% (*n*=54) became pregnant. Similarly, 73.15% (*n*=79) of individuals who wanted to delay pregnancy, were pregnant by the end of the study. Only 26.85% of the women who said they did not want to become pregnant immediately were able to achieve their intended goal (*n*=29). There was not a significant difference between groups (*P*=.057).

Lansdale. Pregnancy intentions and outcomes among young married women in Nepal. Am J Obstet Gynecol MFM 2024.

Table 2 shows the frequency of various pregnancy-related characteristics by survey round. Contraception use among individuals not currently pregnant increased with each round with 36% of women using a method in Round 4. As time went on, women's intentions on delaying pregnancy decreased, which is not surprising. The number of women with unintended pregnancies among those who wanted to delay at baseline versus those who wanted to delay the previous round were similar. By Round 4, there was a greater number of women who disagreed with the idea that contraceptive use is wrong. In Round 1, only 78.26% disagreed with this idea, while 91.86% disagreed with Round 4.

**Table 2:** Frequency of pregnancy-related characteristics by survey round

	Round 1 (*N*=184)		Round 2 (*N*=176)		Round 3 (*N*=176)		Round 4 (*N*=172)	
	No.	%	No.	%	No.	%	No.	%
**Ever pregnant**	NA	NA	79	42.9	125	71.0	134	77.9
**Currently using contraception among individuals not currently pregnant**	41	22.3	38	21.6	35	19.9	62	36.0
**Pregnancy intention among those not currently or ever pregnant** (not right away)	116	63.0	57	32.4	30	17.1	10	5.8
**Unintended pregnancy among those who wanted to delay at baseline**	NA	NA	44	25.0	29	16.5	7	4.1
**Unintended pregnancy among those who wanted to delay the previous round**	NA	NA	44	25.0	20	11.4	7	4.1
**Contraceptive use to delay pregnancy is wrong**								
Agree	33	18.0	21	12.0	12	6.9	10	5.9
Neutral	7	3.8	4	2.3	3	1.7	4	2.3
Disagree	143	78.2	150	85.7	160	91.4	157	91.8

Compared to individuals who intended to delay becoming pregnant, the odds of becoming pregnant were 3.16 times higher for women who intended to become pregnant immediately (OR, 3.16; 95% CI, 2.12–4.70) (Table 3). After adjusting for covariates, the odds of becoming pregnant increased to 4.03 times higher for women who said they wanted to have a child right away (OR, 4.03; 95% CI, 2.51–6.48) (Table 3) compared to those that wanted to delay. Both associations were significant at an alpha level of <0.001. In the sensitivity analysis where we used logistic regression models to examine the association of pregnancy intention at baseline on pregnancy, we found odds ratios of a slightly lower magnitude but the same direction (Table 3). This suggests that the association between pregnancy intention and pregnancy remained consistent.

**Table 3:** Unadjusted and adjusted mixed effects and logistic regression models exploring the association of pregnancy by pregnancy intention (odds ratio, 95% confidence interval)

	*N*	Univariate odds ratio (95% CI)	*P-*value	*N*	Adjusted odds ratio (95% CI)	*P*-value
**Mixed effects logistic regression models**						
Pregnancy intention (ref=not right away)	183	3.16⁎⁎ (2.12–4.70)	<.001	165	4.03⁎⁎ (2.51–6.48)	<.001
**Logistic regression models**						
Pregnancy Intention (ref=not right away)	171	2.20 (0.35–5.02)	.060	154	2.78⁎ (1.03–7.53)	.044

Adjusted for participant's age at baseline, participant's education, birth country, caste, religion, baseline wealth, whether the participant's family paid for a wedding dowry during marriage (yes or no), partner's age, partner's education, whether the participant lives with their spouse (yes or no), participant's age at wedding, marriage type (love or arranged), participant's paid work in last year (yes or no), and empowerment.

⁎ *P*<0.05

⁎⁎ *P*<0.001.

## Comment

### Principal findings

Our findings suggest pregnancy intentions influence pregnancy. After adjusting for confounding, intending to become pregnant immediately was significantly associated with increased odds of becoming pregnant among young women in Nepal in our study. More importantly, more than 70% of the women who did not intend to become pregnant right away, ultimately became pregnant during the two-year period. This suggests that women in this study in Nepal may not have all the resources necessary to prevent or delay pregnancies. Despite a desire to delay, family planning use remained low.

### Results in the context of what is known

Understanding how fertility intentions change and how intentions are related to pregnancy is still an area in need of research, especially among young newly married women in South Asia where there has traditionally been a rapid transition to the first birth after marriage. Current literature is limited by cross-sectional data or longitudinal data with only 2 time points, reducing the ability to measure changing intentions.^15^ Past studies in the 1990s in India that used two measures (baseline and follow up a few years later) found that a half to two thirds of women were not able to delay/avoid pregnancy as desired; contraceptive and childbearing intentions combined here found to be the best predictor of success.^19^^,^^20^ A similar study in India in 2012 that focused on all women (not only young nulliparous women) and had two time points for analysis, found no impact of intentions on pregnancy.^21^ In contrast, we find that intention to have a birth soon is predictive of birth, however, a great many women do not have their desires met. By comparing analyses that used a stable baseline measure and looked at outcomes 2 years later, and analysis of a time-varying indicator of intention, we find similar results, however, the time-varying variable shows a stronger association. This suggests that while capturing time variation in intentions may be important, at least in this short time frame, the additional information gleaned is not substantial. Past literature has found that the desire for male children or sons influences fertility goals, however, our study is free from this bias since no participants had given birth before the start of the study.^22^

Furthermore, social, structural, and clinical factors play a part in both intentions and outcomes. Women with the intention of delaying pregnancy were more educated, richer, had more educated husbands, and were more likely to engage in paid employment. Nepalese demographic data has shown that richer and more educated women are less likely to experience unintended pregnancies.^23^ In addition, women who intended to delay their pregnancy reported a higher sense of empowerment. This finding is consistent with previous evidence suggesting that social and structural forms of empowerment may contribute to a woman's decision to delay or prevent pregnancy.^24^ Fertility norms, which are likely associated with empowerment, are also associated with fertility intentions in India.^25^

### Clinical implications

By the end of the study, most women agreed with the idea of using contraceptive methods, though many had never used a method. Of the women who did not intend on pregnancy right away, only 22.3% had ever used a contraceptive method at baseline. We do not know the reasons for the disconnect between desire to delay and use of contraception, although prior research found lack of communication between spouses, knowledge, stigma on infertility, and empowerment/agency of both men and women contributed to low use.^12^

### Research implications

More research is needed on pregnancy intentions using longitudinal data where pregnancy intention is assessed prospectively in other settings and using larger samples. Future studies could incorporate measures of the strength of pregnancy desire or desire to delay, for example using the Desire to Avoid Pregnancy Scale.^26^ By better understanding this relationship, interventions and policies could be introduced to strengthen, educate, and empower women. Future research should also focus on contraceptive access and barriers as these are critical in allowing individuals to make reproductive decisions.

### Strengths and limitations

The findings from this study are significant because most evidence examining pregnancy intentions is from cross-sectional studies. This study used longitudinal data to see if pregnancy intentions at baseline were associated with pregnancy at a future timepoint. In addition, this study adjusted for many important confounders including living with spouse, working, caste, wealth, and empowerment. However, it is important to acknowledge the limitations of this analysis. This study has a small sample size of 200 women who were only eligible to participate if they were newly married and lived with their mothers-in-law. In addition, the women lived in one district of Nepal. Therefore, the generalizability of these findings is limited. While focusing on young women limits generalizability to older women, this is also a strength and contribution, since there is less evidence about this population. Although this study is longitudinal, the duration is only two years which limits the long-term implications. Since women's pregnancy intentions could change with time, it could be beneficial to examine this relationship in a longer study. In addition, this study used self-report measures and some women might not have known they were pregnant and then lost the baby before the next survey, which could bias the results.

## Conclusions

Despite intending immediate pregnancy being predictive of newly married young women in Nepal becoming pregnant, over 70% of women who said they wanted to delay had given birth 2 years later. This suggests that women in this setting experience challenges in meeting their reproductive goals. Helping young, newly married women be able to delay their first birth should be a focus of reproductive health programming in this setting.

## CRediT authorship contribution statement

**Aimee J. Lansdale:** Writing – original draft, Writing – review & editing, Visualization, Methodology, Formal analysis. **Mahesh C. Puri:** Writing – review & editing, Project administration, Data curation. **Nadia Diamond-Smith:** Writing – review & editing, Visualization, Validation, Supervision, Resources, Project administration, Investigation, Funding acquisition, Formal analysis, Conceptualization.
